# Photocurrent Generation by Plant Light-Harvesting Complexes is Enhanced by Lipid-Linked Chromophores in a Self-Assembled Lipid Membrane

**DOI:** 10.1021/acs.jpcb.4c07402

**Published:** 2025-01-09

**Authors:** Masaharu Kondo, Ashley M. Hancock, Hayato Kuwabara, Peter G. Adams, Takehisa Dewa

**Affiliations:** † Department of Life Science and Applied Chemistry, Graduate School of Engineering, Nagoya Institute of Technology, Gokiso-cho, Showa-ku, Nagoya 466-8555, Japan; ‡ School of Physics and Astronomy, University of Leeds, Leeds LS2 9JT, U.K.; § Astbury Centre for Structural Molecular Biology, University of Leeds, Leeds LS2 9JT, U.K.; ∥ Department of Nanopharmaceutical Sciences, Nagoya Institute of Technology, Gokiso-cho, Showa-ku, Nagoya 4668-8555, Japan

## Abstract

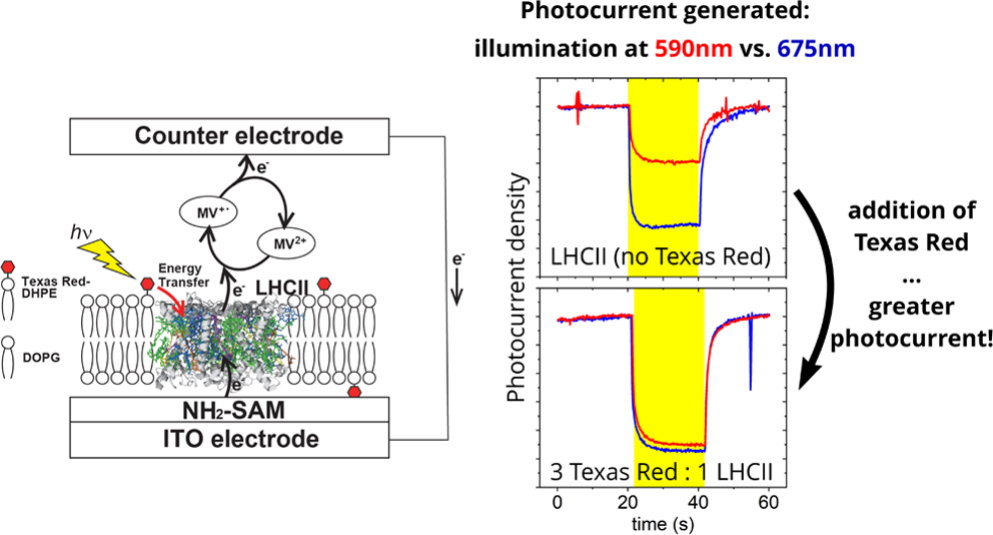

The light-harvesting pigment–protein complex II (LHCII) from plants can be used as a component for biohybrid photovoltaic devices, acting as a photosensitizer to increase the photocurrent generated when devices are illuminated with sunlight. LHCII is effective at photon absorption in the red and blue regions of the visible spectrum, however, it has low absorption in the green region (550–650 nm). Previous studies have shown that synthetic chromophores can be used to fill this spectral gap and transfer additional energy to LHCII, but it was uncertain whether this would translate into an improved performance for photovoltaics. In this study, we demonstrate amplified photocurrent generation from LHCII under green light illumination by coupling this protein to Texas Red (TR) chromophores that are coassembled into a lipid bilayer deposited onto electrodes. Absorption spectroscopy shows that LHCII and lipid-linked TR are successfully incorporated into lipid membranes and maintained on electrode surfaces. Photocurrent action spectra show that the increased absorption due to TR directly translates into a significant increase of photocurrent output from LHCII. However, the absolute magnitude of the photocurrent appears to be limited by the lipid bilayer acting as an insulator and the TR enhancement effect reaches a maximum due to protein, lipid or substrate-related quenching effects. Future work should be performed to optimize the use of extrinsic chromophores within novel biophotovoltaic devices.

## 1 Introduction

The pigment–protein complexes found in natural photosynthesis can be extracted from biological organisms and interfaced with inorganic photovoltaic devices, in an attempt to improve the effectiveness of solar energy capture. Several studies have shown that plant Photosystems (PSI, PSII) and bacterial Reaction Center (RC) complexes can act as “photosensitizers” when they are deposited onto electrodes, increasing the overall photocurrent generated.^1−4^ However, these complexes are susceptible to photooxidative damage and degradation over time. Light-Harvesting (LH) antenna complexes are an interesting alternative material that could have a few advantages over reaction-center-type complexes. For example, the major antenna protein from spinach, LHCII, is (i) relatively robust,^5^ (ii) one of the most abundant membrane proteins on the planet,^6^ and (iii) of interest for its photoprotective properties.^7^ Ishigure et al. first reported that LHCII from spinach produces a photosensitizing effect.^8^ Since then, some groups, including ours, have investigated the photocatalytic function of LHCII aiming toward biohybrid solar cells and hydrogen production.^9−15^ In one example, spinach LHCII was electrostatically adhered to indium tin oxide (ITO) electrodes and found to produce a photocurrent under illumination with blue or red light.^13,14^ The finding that LHCII could generate a photocurrent was unexpected because under natural biological conditions these complexes transfer excitation energy between each other but they do not ever transport electrons. In photovoltaic devices, it is possible that electron transfer was induced under the conditions that LHCII were exposed to: possibly the large applied electric potential.^13^ The authors of the latter study proposed that LHCII could export electrons from the terminal (lowest-energy) cluster of chlorophylls. In the current paper, we extend the usage of plant LHCII as a sensitizer of photovoltaic devices, by interfacing it with extrinsic chromophores.

It is important to consider how proteins can be physically interfaced with the electrode surface. Many studies generating photocurrent from PS/LH complexes have coupled the purified proteins onto electrode surfaces via electrostatic or covalent bonding,^1,13^ however, this is very different to the natural environment for a membrane protein and is likely to destabilize the protein complex. Lipid bilayers are the natural matrix which holds all transmembrane proteins in place, including PS/LH complexes. Supported lipid bilayers (SLBs) are an established model membrane system that have been studied to understand the biophysics of membranes.^16−18^ SLBs will spontaneously self-assemble to generate a microscale thin film on solid surfaces and have the advantage that they stabilize membrane proteins in their natural orientation.^19,20^ Considering again photovoltaic devices, choosing to employ SLBs containing PS/LH complexes as a film on the electrode can allow and promote the more effective association of the protein with the electro-active surface. Recently, we have reported that lipid bilayers containing bacterial RC and LH complexes, on an ITO electrode, are effective at producing photocurrent.^21−23^ However, plant LHCII has never before been investigated in a lipid membrane environment for the purpose of photocurrent generation.

A number of previous studies have shown that extrinsic chromophores can enhance the magnitude of the photocurrent generated by bacterial RC-LH1 protein complexes.^21,22,24^ These extrinsic chromophores, such as ATTO and Alexa, were chosen to be spectrally complementary to the protein complex and acted to fill a region of the visible spectrum where there was low absorption strength for the natural RC-LH1 complex, transferring additional excitation energy toward the RC via Förster resonance energy transfer (FRET). Other studies have demonstrated that the absorption strength of peripheral antenna LH complexes such as bacterial LH2 and plant LHCII can be enhanced with extrinsic chromophores in a similar manner. Instead of cross-linking external chromophores to pigment–protein complexes, a self-assembling strategy using lipid bilayers is one of the potential architectures for efficient excitation energy transfer.^25,26^ Particularly, synthetic lipid-linked chromophores have recently found utility as excitation energy donors that are effective at energy transfer to LH protein complexes with a few advantages.^27−29^ Lipid-linked chromophores were shown to readily self-assemble with other lipids and membrane proteins into an organization that brings the chromophores into close contact with the desired LH complexes without the requirement for covalent cross-linking,^30−32^ or genetic modification.^33^ This noncovalent, lipid-based self-assembling approach can be considered to be simpler and more flexible than other methods^28^ and it allows researchers to choose any protein-of-interest and any lipid-linked chromophore and to assemble them in a modular fashion.^29^ Many different conjugates between lipids and chromophores are commercially available and are ready for use in such systems.^34^ In the current paper, we attempt to quantify the effectiveness of using a lipid-linked Texas Red (TR) chromophore that associates with plant LHCII and show that this combination produces much greater photocurrent than either LHCII or TR in isolation.

## 2 Materials and Methods

### 2.1 Materials

Tris(hydroxymethyl)aminomethane (Tris) was obtained from Sigma-Aldrich. Methyl viologen was purchased from Tokyo Kasei Co. Ltd. Surfactants, *n***-**dodecyl-β-d-maltoside (β-DDM) and *n*-octyl-β-d-glucopyranoside (β-OG) were obtained from Dojindo. Texas Red linked to a dihexadecanoyl-*sn*-glycero-3-phosphoethanolamine lipid (TR-DHPE) was purchased from Thermo Fisher Scientific. 1,2-dioleoyl-*sn*-glycero-3-phospho-(1′-*rac*-glycerol) (DOPG) was a gift from Nippon Fine Chemical Co., Ltd. Triton X-100 and KCl were obtained from FUJIFILM Wako Pure Chemical Co. Ltd. The indium tin oxide electrode (sheet resistance ≤5 Ω/sq) was purchased from Geomatec.

### 2.2 Isolation of the LHCII from Spinach

LHCII trimer was isolated from spinach leaves and purified as previously described.^35,36^ Thylakoid membranes were collected from spinach by centrifugation at 19,000 *×g* and 4 °C for 10 min. The LHCII trimer was solubilized from thylakoid membranes by the addition of 1% Triton X-100 (v/v) in a Tris-HCl buffer solution (20 mM, pH 8.0). The LHCII trimer was then separated from Photosystem I by sucrose density centrifugation at 105,000 *×g* and 4 °C for 16 h. The LHCII trimer was obtained as a single band in the sucrose solution. The LHCII trimer was poured into a solution of 100 mM KCl and 10 mM MgCl_2_ to precipitate. The precipitate was collected by centrifugation at 2460 *×g*. The resulting precipitate LHCII trimer was solubilized in 50 mM Tris-HCl buffer (pH 7.4) containing 0.03 wt % β-DDM. LHCII trimer was characterized by ultraviolet–visible (UV–Vis) absorption and static fluorescence spectroscopy.

### 2.3 Reconstitution of LHCII into Lipid Bilayers

DOPG and TR-lipid were dissolved in chloroform and mixed in the desired ratios. After weighing the required amount of lipid in a glass vial, a stream of nitrogen gas was blown while forming a lipid thin film on the glass surface. The obtained lipid film was subjected to vacuum drying for at least 6 h and stored at −20 °C until further use. The lipid film was hydrated at room temperature for 30 min by adding 50 mM Tris-HCl buffer (pH 7.4), resulting in the formation of multilamellar vesicles (MLVs). To prepare the lipid and detergent comicelles, 10 wt % β-OG dissolved in Milli-Q water was added to the MLVs to achieve a final concentration of 0.78 wt %. The mixture was thoroughly vortexed and then allowed to stand at room temperature for 1 h. The solubilized LHCII with 0.03 wt % β-DDM, was mixed with comicelles to achieve a lipid-to-protein ratio (L/P ratio) of 250 (molar/molar). The mixture was incubated at 0 °C in the dark for 30 min. The final concentration of the lipid was adjusted to 1 mM, and the final concentration of the LHCII trimer was set to 4.0 μM. After a 1 h incubation, Bio-Beads were added to the solution at a minimum necessary amount of 2–3 times the weight of the sample. The mixture was gently mixed at 4 °C in the dark for 18 h (overnight). After stirring, the solution was separated from the Bio-Beads using a micropipette, and the LHCII proteoliposome solution was transferred to a 1.5 mL plastic tube and kept on ice. These proteoliposome solutions (reconstituted LHCII in lipid bilayers) were stored at 4 °C, never frozen, and all measurements were performed on samples within 1 week after reconstitution.

### 2.4 Assembly of LHCII Proteoliposomes onto ITO Electrodes

An ITO electrode (2 cm^2^) was incubated in an ethanol solution of 10 mM 6-amino-1-hexanethiol for 12 h at room temperature to form a self-assembled monolayer (SAM). The electrode was carefully rinsed with ethanol and blown dry with nitrogen. An aliquot of LHCII proteoliposome solution (20 μL) was placed onto the amino group-modified SAM-ITO electrode to form a SLB containing LHCII. After incubating at 4 °C in the dark for 12 h, the ITO electrode was washed with 50 mM Tris-HCl buffer (pH 7.4). The concentration of LHCII immobilized on the ITO electrode was determined using the Lambert–Beer law based on the absorbance at *Q*_*y*_ band of Chl *a*. The obtained concentration in mol/L was converted to a two-dimensional concentration in *m*^2^ (*m*^3^ → *m*^2^) and the unit was changed to pmol cm^–2^.^37^

### 2.5 UV–Vis Absorption Spectroscopy

UV–visible absorption spectra were recorded with a U1800 Shimadzu spectrophotometer. The ITO electrodes covered by LHCII-containing SLB were placed perpendicular to the light beam in a glass cuvette. A baseline was recorded with the ITO electrode without the LHCII SLB.

### 2.6 Photocurrent Measurements

Photocurrents were measured at −0.2 V vs Ag/AgCl in an electrochemical cell that contained three electrodes: a LHCII-SLB modified ITO electrode as a working electrode, an Ag/AgCl (saturated KCl) as a reference electrode, and a platinum wire as a counter electrode. The working electrode was illuminated with a xenon lamp unit (SM-25, Bunkokeiki, Japan), through a monochromator. Photocurrent response data were recorded with a HZ-5000 potentiostat (Hokuto Denko, Japan) The solution consisted of 0.1 M phosphate buffer (pH 7.5), containing 0.1 M NaClO_4_ and 10 mM methyl viologen as an electron acceptor. The irradiation intensity (about 1.2 mW/cm^2^) was similar at every illumination wavelength. In the wavelength dependence measurement of the photocurrent, the intensity of PFD (photon flux density) was set at 59–74 μmol/m^2^ s (60.9 μmol/m^2^ s at 590 nm and 67.3 μmol/m^2^ s at 675 nm).

### 2.7 Fluorescence Spectroscopy

Fluorescence excitation and emission spectra were recorded with FluoroMax-4-NS (HORIBA). Before fluorescence measurements, the proteoliposome solution was diluted with 50 mM Tris-HCl buffer (pH 7.4) to an absorbance of 0.1 at 675 nm (*Q*_*y*_ band of Chl *a*) in a quartz cell with 10 mm of path length. The excitation and emission wavelengths used are noted in the figure captions. Time-resolved fluorescence measurements were recorded using the Nanofinder30 (Tokyo Instruments) with a streak camera (C10627–03, Hamamatsu Photonics), either for proteoliposomes solutions in cuvettes or for membranes deposited onto ITO surfaces. A 509.4 nm picosecond pulse diode laser (PIL051X, Advanced Laser Diode Systems) was used as the excitation source. The time-correlated single photon counting data was plotted as decay curves and the fluorescence lifetimes were extracted from fitting the curve to multiexponential decay function.

## 3 Results and Discussion

### 3.1 Concept for Photocurrent Enhancement with a Lipid-Linked Chromophore and Workflow of the Approach

**Figure 1:**
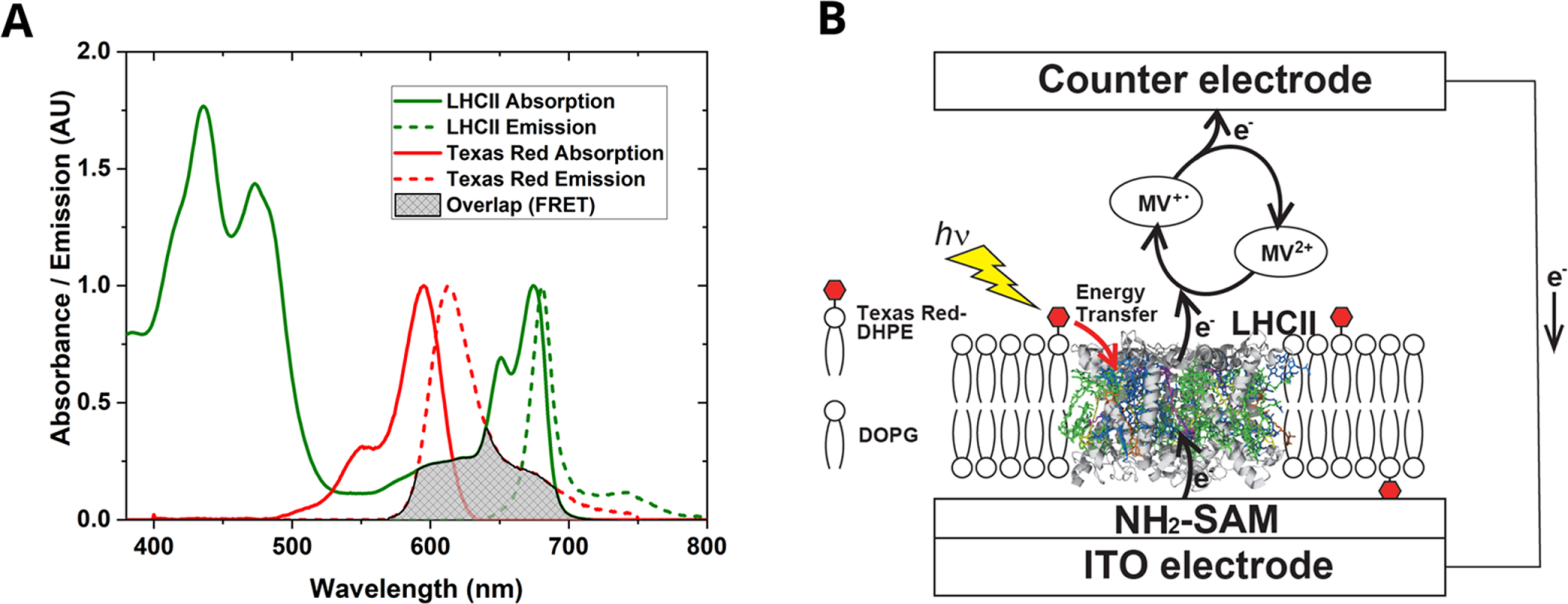
Spectra and cartoon of the overall concept for using lipid-linked chromophores to enhance photocurrent generated by LHCII protein complexes. (A) Absorption and fluorescence spectra, as labeled, demonstrating the potential for energy transfer from TR to LHCII. The spectral overlap between the fluorescence of TR and the absorption of LHCII represents the energetic coupling and possibility of TR → LHCII FRET. (B) Cartoon of the processes occurring in the system. Photon absorption by a TR chromophore (lightning bolt) and excitation energy transfer to LHCII (red arrow) leads to enhanced electron flow (black arrows) between the electrodes. The electrochemical circuit is completed by the mediator methyl viologen (MV^+•^/MV^2+^).

We previously demonstrated a method of utilizing lipid-linked chromophores, which diffuse freely and disperse homogeneously within lipid bilayers, to enhance the spectral range for photon absorption of LH proteins.^28,29^ This was accomplished by coreconstitution of the membrane proteins and chromophore molecules into self-assembled lipid bilayers. The chromophore TR was established to absorb green light (the spectral region where LHCII has limited absorbance) and act as an efficient donor of excitation energy to the chlorophylls within LHCII leading to an enhancement of its fluorescence by up to 300%. It is well-known that SLBs (containing proteins) can be generated on suitably hydrophilic flat surfaces and we have previously shown that proteoliposomes containing LHCII and TR-DHPE can be used as starting material to generate high quality membranes on solid substrates, with the TR-to-LHCII energy transfer conserved in these SLBs.^29^ In the current work, we conceived that TR-to-LHCII energy transfer (Figure 1A) on a two-dimensional (2D) surface could be applicable to electrodes for photocurrent generation (Figure 1B). For this to be successful, the increased number of excited states reaching LHCII due to energy transfer from TR would need to translate into elevated rates of electron transfer between LHCII and the electrodes.

In order to test this hypothesis, we utilized an ITO-based photovoltaic system previously demonstrated to work effectively with LH proteins incorporated within SLBs acting as sensitizers.^21−23^ The ITO surface was modified via established silane surface chemistry to display amino groups which provide positive charges and sufficient hydrophilicity for SLBs to be formed. Lipid vesicles containing LHCII were incubated with the surface to create an SLB with reconstituted LHCII in a native-like environment (see Materials and Methods section). To determine whether TR was effective in enhancing photocurrent generation in the system, a predetermined amount of TR-DHPE was included in several samples to give a range of TR-to-LHCII ratios. A statistically significant number of TR chromophores were expected to be close enough to LHCII so that excitation energy transfer would be efficient. Note that TR-to-TR excitation transfer would be possible, allowing for an even greater network of TR to be connected to LHCII by multiple steps of exciton hopping.^27^ Electron flow between LHCII and the electrode could be induced either after direct excitation of the chlorophyll (Chl) or carotenoid (Car) pigments within LHCII^14^ or by the indirect excitation of these same pigments after photon absorption by TR and FRET to pigments within LHCII. This indirect route is our main interest in the current study, as shown in the cartoon in Figure 1B. Methyl viologen (MV^2+^) takes the role of an electron acceptor and carrier that acts to transport electrons between the protein and the counter electrode. We assume that the positive charge left on the LHCII is neutralized by injection of an electron from the ITO (equivalently: holes are transported from the LHCII complex to the ITO electrode). In order to fabricate this biophotovoltaic system, the first step was to assemble a stable vesicular form of the proteolipid membranes that contained the LHCII protein complex and the lipid-linked TR molecules and the second step was to deposit them onto the electrode surface.

### 3.2 Assembly of Proteoliposomes and Formation of Membranes on Electrodes

In order to generate appropriate material for attachment to the electrodes, a series of proteoliposome samples were prepared that contained three components: (i) the LHCII protein complex, (ii) normal DOPG lipids, and (iii) a range of concentrations of TR-DHPE. We aimed to produce vesicles containing a standard quantity of LHCII and normal lipids (LHCII/DOPG ratio of 1:250 mol/mol) and varied the amount of TR-DHPE included from 0.28 to 1.71% TR-DHPE relative to DOPG lipids (mol/mol). These were compared with a simple control sample of liposomes containing 1.0% TR-DHPE/DOPG without any LHCII. The negatively charged phospholipid DOPG was chosen as the bulk lipid for liposomes because it has been previously shown to be effective at forming a planar lipid membrane on 6-amino-1-hexanthiol-modified ITO electrodes via electrostatic interactions.^23^

**Figure 2:**
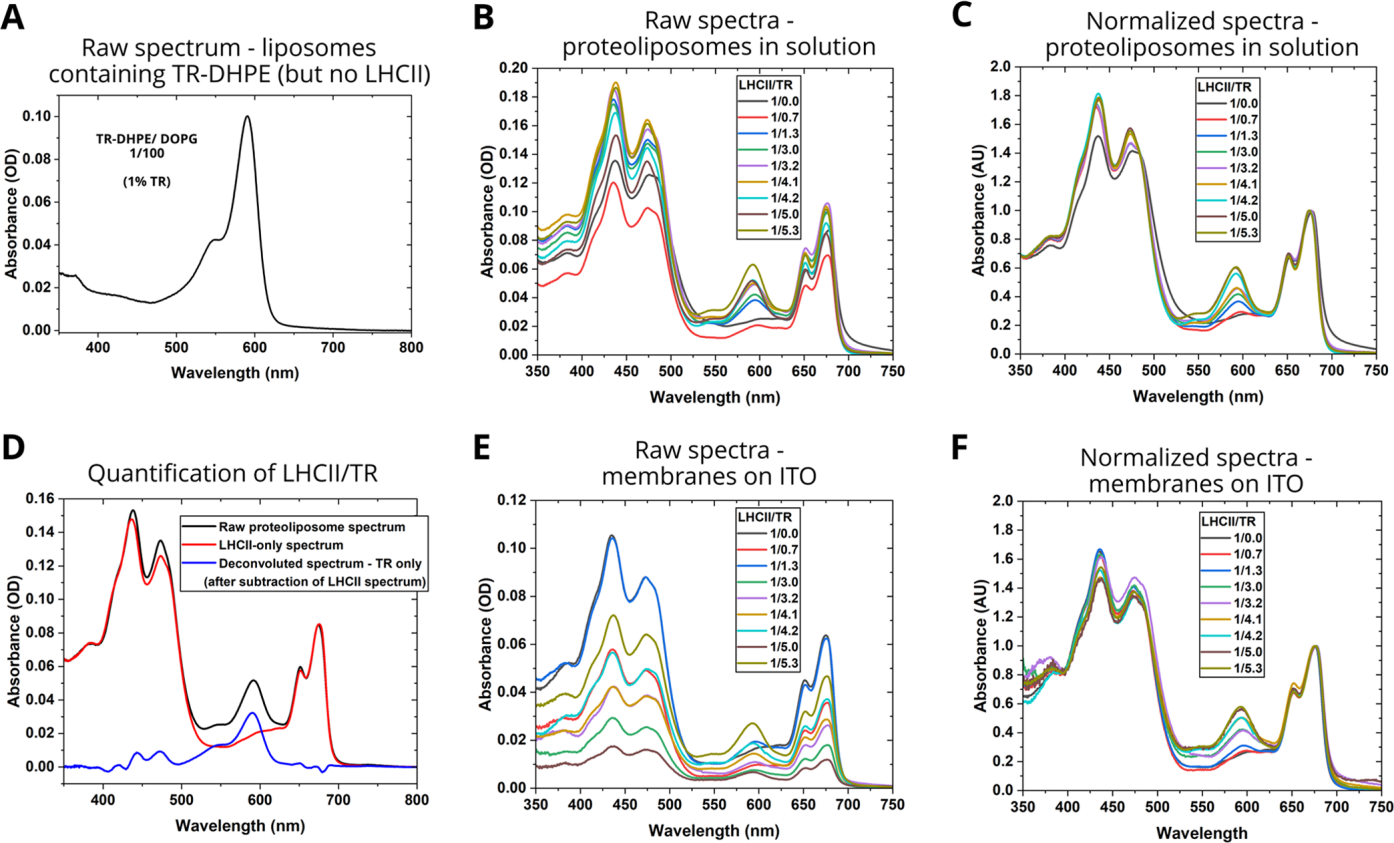
Absorption spectra of proteoliposome samples for analysis of the LHCII and TR content. (A) Absorption spectrum of TR-DHPE/DOPG liposomes (1:100 mol/mol) in aqueous solution. (B) Raw absorption spectra of LHCII/TR proteoliposomes in aqueous solution. All vesicles were prepared with an LHCII/DOPG ratio of 1:250. (C) Normalized absorption spectra of LHCII/TR proteoliposomes in solution, adjusted to a height of 1.0 at 675 nm (Chl *aQ*_*y*_ peak). (D) Example graphs showing the process of spectral deconvolution, as used for determining the quantities of LHCII and TR-DHPE within membranes, to generate Tables S1 and S2. (E) Raw absorption spectra of membranes after assembly onto ITO electrodes. (F) Normalized absorption spectra of membranes after assembly onto ITO electrodes, adjusted to a height of 1.0 at 675 nm. For simplicity and comparability, the LHCII/TR ratio shown in all panels refers to the actual TR/LHCII ratio achieved in the membranes assembled onto the ITO electrodes, as calculated in Table S2.

In order to assess the composition of the membranes that were formed by this procedure, absorption spectroscopy was performed on solutions of the proteoliposomes. These spectra showed that there was successful incorporation of both LHCII and TR from the presence of their characteristic peaks (Figure 2A,B). The bands related to LHCII were observed at relatively consistent wavelengths for all samples, with peaks between 400–500 nm representing the overlapping Chl Soret and Car absorption, the peak at 650 nm representing the Chl *bQ*_*y*_ transition and the peak at 675 nm representing the Chl *aQ*_*y*_ transition (Figure 2B). This confirmed that the protein retained its usual pigment composition, in all cases, after incorporation into lipid bilayers. The sample series was designed to contain a range of TR and, indeed, the height of the peak at ∼590 nm which related to TR absorption increased as expected (Figure 2C versus Figure 2A). For later analyses it was important to know the exact composition of the membranes, however, one cannot assume that all material included in the starting mixture actually assembles into the membranes because the yield of incorporation of the protein and TR into lipid membranes can vary. Therefore, for each of these samples, the actual molar TR-to-LHCII ratio achieved in the vesicles was calculated from the magnitude of the peaks in absorption spectra, by using the known molar absorption coefficients, after deconvolution of the peaks (see Figure 2D and Table S1). The LHCII concentration achieved was found to be relatively consistent, with calculated concentrations between 2.08–3.16 μM, and TR incorporation into liposomes had the desired wide range of concentrations, calculated as 2.84 to 17.05 μM. This led to a range of LHCII/TR mole-to-mole ratios being tested from 1:1.4 to 1:5.6 (see Table S1 for calculations). Further analysis of spectra confirmed that liposome-reconstituted LHCII remained intact and functional with minimal peaks shifts in absorption, fluorescence excitation and fluorescence emission spectra compared to isolated LHCII (see Figure S1).

The next stage was to generate membranes on electrode surfaces. Therefore, each proteoliposome solution was incubated with a series of amino-modified ITO electrode surfaces to form SLBs. Absorption spectra were acquired on the assembled devices to assess the LHCII protein and TR chromophore content within these SLBs (Figure 2E). It was clear that the membranes had assembled successfully and that a wide range of TR/LHCII ratios had been maintained, from the range of heights of the TR peak at ∼590 nm and the relatively similar LHCII peaks between 400–500 and 650–700 nm (Figure 2F compared to Figure 2C). More detailed analysis showed that there was significant sample-to-sample variability in the amount of material adsorbed onto the electrode surfaces but, importantly, the TR-to-LHCII range of the membranes was similar before and after assembly on ITO (see Table S2 for calculations). We expect that this variation in the amount of material attached to the ITO substrates was due to the “sticky” nature of the stromal side of LHCII, which interacts with each other between lipid layers via electrostatic interactions.^38^ This may result in the assembly of multilayers onto ITO that are challenging to control. One goal of future work is to improve the consistency of membrane deposition onto ITO substrates. The measured amount of LHCII deposited onto the electrodes (within SLBs) varied from 7.0 to 36.8 pmol/cm^2^, broadly consistent with previous studies that used detergent-solubilized LHCII.^13^ The amount of TR on the electrodes was estimated to be 14.3–145.4 pmol/cm^2^, giving a broad range of LHCII/TR ratios for testing the photocurrent performance. We determined that this set of devices would allow the effect of TR-to-LHCII ratio to be assessed in subsequent photocurrent analyses, but that the signal from TR must be normalized relative to the signal from the LHCII protein in order to allow a quantitative comparison. The TR/LHCII ratios calculated on ITO ranged from 1:0.7 to 1:5.3 (Table S2) and these values are used when referring to samples henceforth.

### 3.3 Photocurrent Response of LHCII/Lipid Membranes Containing a Range of Concentrations of Lipid-Linked Texas Red

**Figure 3:**
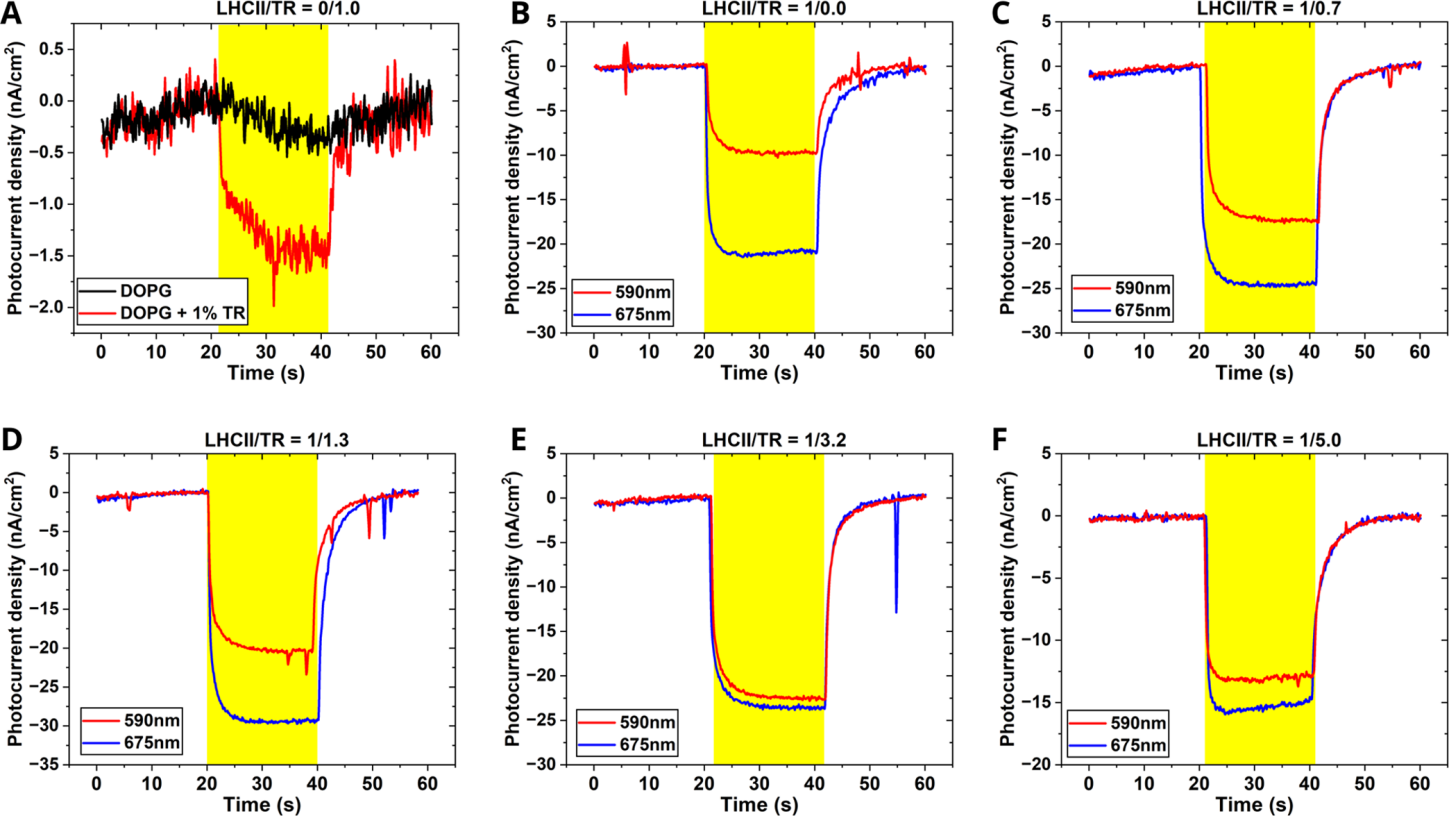
Photocurrent response of devices containing membranes assembled onto amino-modified ITO electrodes. (A) Photocurrent response of lipid membranes either containing TR (red) or without TR (black), under illumination at 590 nm, from the sample shown in Figure 2A. (B–F) Photocurrent response of LHCII-TR membranes, from the samples shown Figure 2E,F. The photocurrent response was monitored under illumination at either 590 nm (red) or 675 nm (blue). The yellow-colored time regions represent the illuminating period. The electrochemical cells had an electrolyte solution of 0.1 M phosphate buffer (pH 7.5) containing 0.1 M NaClO_4_ and 10 mM methyl viologen.

Next, the photocurrent generated by this series of biomembrane-enhanced devices was quantified and compared. The first “negative control” sample of lipid membranes without any protein or TR showed a photocurrent of approximately zero (a featureless noisy line, Figure 3A, black line). The second negative control sample of lipid membranes containing TR-DHPE but without any proteins showed a very low peak photocurrent density of only −1.5 nA/cm^2^ (Figure 3A, red line). For the range of LHCII-containing samples, the photocurrent responses were assessed using two different illumination wavelengths that were designed to selectively target the LHCII Chl pigments (675-nm illumination) or mainly target the TR chromophores (590-nm illumination). The magnitude of the maximum photocurrent density that was generated by 675-nm LHCII illumination varied from −15 to −30 nA/cm^2^ between samples (Figure 3B–F, blue traces), according to the different quantities of membranes adsorbed onto ITO, as noted earlier. Therefore, it was most instructive to compare the ratio of photocurrents generated with the two alternate illumination wavelengths (Figure 3B–F, red vs blue traces). For LHCII membranes in the absence of TR (Figure 3B), the maximal photocurrent generated with 590-nm illumination was less than 50% of the max photocurrent with 675-nm illumination, due to the low absorption from LHCII in this range. The photocurrent density with 590-nm illumination relative to the level with 675-nm illumination was found to increase substantially with increasing TR-to-LHCII ratio (Figure 3B–F). This increase was consistent with the trend observed in fluorescence excitation spectra of LHCII-TR liposomes, where higher concentrations of TR resulted in an increased transfer of energy to LHCII when excited in the 550–600 nm region (see Figure S2 and Table S3). The finding that TR-lipids are effective at generating photocurrent when combined with LHCII but ineffective when on their own (Figure 3A versus 3B–F) suggests that TR-DHPE does not work effectively in directly transporting electrons to an electrode, but it instead works like an additional light-harvesting pigment for LHCII which generates the photocurrent. In this way, TR indirectly increases the photocurrent generated by augmenting the light-harvesting capability of LHCII.

We note that the photocurrent generation is lower than reported in our previous study using detergent-solubilized LHCII^13^ and this could be attributed to slight differences in experimental conditions (lower illumination intensity) and/or the known effect of LHCII self-quenching when reconstituted into lipid bilayers compared to when isolated in detergent.^12,28,39−41^ The level of photocurrent generation is also lower than reported in previous studies using RC proteins reconstituted into SLBs^22^ immobilized onto ITO electrodes, as may be expected because RCs function in nature as an electron transporter whereas LHCII does not. The advantages and disadvantages of the LHCII-SLB architecture are discussed later.

### 3.4 Action Spectra Revealing How the Illumination Wavelength Affects Photocurrent Generation

**Figure 4:**
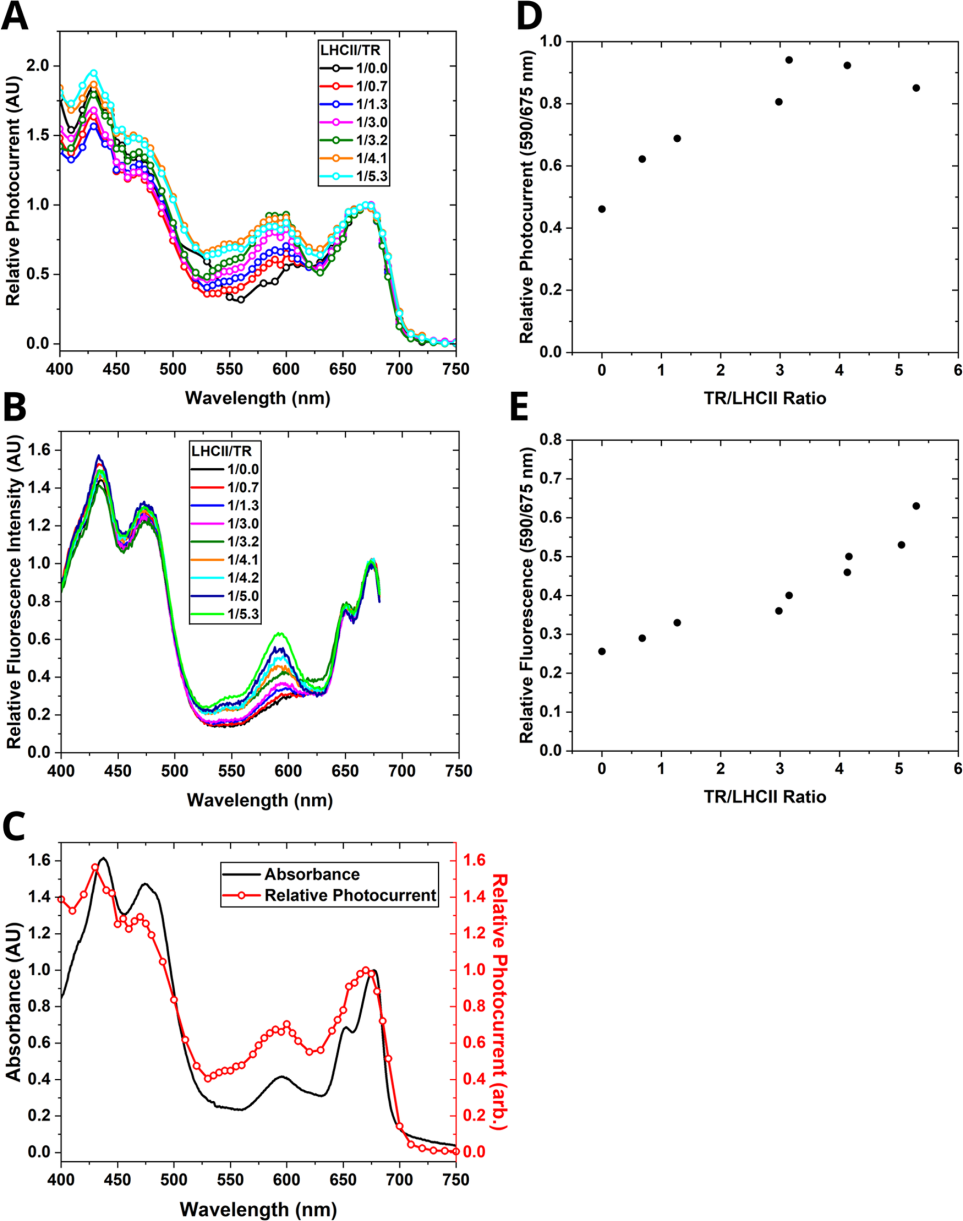
Analysis of photocurrent action spectra versus fluorescence spectra. All spectra were normalized to 1.0 at a wavelength of 675 nm. (A) Normalized photocurrent action spectra of LHCII/DOPG and LHCII/TR-DHPE/DOPG membranes on ITO electrodes. (B) Normalized fluorescence excitation spectra of LHCII/TR-DHPE/DOPG membrane vesicles in solution. (C) Comparison between the absorption spectrum and the photocurrent action spectrum of LHCII/TR-DHPE = 1:3.2. (D) Relative photocurrent generated when illuminating at 590 nm (*I*_590_) vs at 675 nm (*I*_675_), as a function of the TR-to-LHCII ratio of the membranes tested, using data from the spectra in panel (A). The raw spectra and tabulated numerical data of all photocurrent measurements is provided in Figure S3 and Table S4, respectively. (E) Relative fluorescence intensity upon excitation at 590 nm (*F*_590_) vs at 675 nm (*F*_675_), using data from the spectra in (B).

To determine the effectiveness of the combination of LHCII and TR, we assessed the spectral dependence of the devices. In order to do this, action spectra were acquired where the photocurrent was measured when illuminating the devices at a range of wavelengths across the visible range, in 15-nm steps. The normalized photocurrent data is shown in Figure 4A. The action spectra in the 400–500 and 650–700 nm ranges match the shape of the LHCII absorption spectrum, showing that excitation of any pigments leads to photocurrent generation, implying that the pigments within LHCII are strongly coupled (black plot in Figure 4A). The peaks at 650 (*Q*_*y*_ band of Chl *b*) and 675 nm (*Q*_*y*_ band of Chl *a*) are poorly separated in the action spectra compared to the absorption spectra, simply due to the lower spectral resolution due to the wide slit width (150 μm, corresponding to 12 nm of FWHM of illumination light) in the spectrometer that is required to generate sufficient signal in photocurrent measurements. The action spectra clearly reveal a peak related to TR between 550–600 nm (Figure 4A), matching its position in absorption and excitation spectra at 590 nm (Figures 4B and S2), and increasing with the TR-to-LHCII ratio. Unexpectedly, the maximal level of photocurrent generated across the TR peak (between 560–630 nm), was similar to the photocurrent generated at the chlorophyll *Q*_*y*_ peak (between 650–680 nm) even though the level of light absorption across this range was much lower (see Figure 4C). This suggests that the combined effect of the TR and LHCII is greater than either component alone. The ratio of photocurrents generated due to TR vs LHCII was quantified and plotted against the membrane composition ratio of TR-to-LHCII, and this revealed a roughly linear trend of increasing photocurrent as LHCII-to-TR ratio increased up to 1:3.2, with similar photocurrent at higher TR-to-LHCII ratios (Figure 4D). This is in contrast to the increasing LHCII fluorescence that was observed for vesicles in solution as the TR-to-LHCII ratio was increased (Figure 4E).

The reasons behind this limitation for photocurrent enhancement due to TR, despite an increasing fluorescence enhancement, were not immediately clear. Therefore, we performed some control fluorescence measurements to attempt to discern this. The fluorescence intensity was expected to relate to the number of excited states of the molecule concerned, so that TR fluorescence should relate to the availability of TR excited states which may be donated to LHCII and LHCII fluorescence should relate to LHCII excited states which may generate a photocurrent. Measurements on TR-DHPE/DOPG vesicles in solution showed that there was a reduction in TR fluorescence as the TR-to-DOPG ratio increased (Figure S4A,B) concurrent with the known effect of self-quenching of TR at high concentrations.^42,43^ Furthermore, measurements of TR-DHPE/DOPG membranes on ITO showed that there is quenching of TR fluorescence due to the electrode surface even in the absence of the electron mediator MV/MV^2+^ (Figure S4C). This suggests that additional nonradiative decay pathways are introduced to TR due to the surface, which could include energy dissipation as heat that we cannot directly observe, in addition to very small amounts of direct electron transfer (TR → MV) that was already observed (Figure S5). It seems that the increase in TR self-quenching observed at higher TR concentrations does not significantly reduce the amount of energy transferred to LHCII, as evidenced by the linear relationship between TR/LHCII ratio and LHCII fluorescence when exciting TR at 590 nm (Figure 4E). This could be explained by the rate of TR-to-LHCII energy transfer^27^ being significantly higher (ps) than the rate of TR-TR self-quenching^43^ (ns). It is also known that the LHCII protein can switch into a quenched state^7,44−46^ but how this would be affected by increasing the concentration of TR is unclear. It is possible that the LHCII protein rearranges when it is deposited onto ITO surfaces^12^ leading to different quenching behavior and a different dependence on TR-DHPE concentration. A final possible explanation for the discrepancy between the linear rise in LHCII fluorescence emission with increasing TR concentration (Figure 4E) and the limitation of photocurrent generation in the same TR-LHCII range (Figure 4D) is an inherent limitation in the electron transfer process but not the excitation energy transfer processes. This could be a limitation in the rate of electron expulsion from LHCII to MV, or a limitation in the rate of electron transfer from the ITO electrode to the LHCII. The molecular mechanism of electron transfer from the electrode to LHCII and its relationship with exciton transfer from TR → LHCII cannot be fully established from our work due to the large number of different transfer and decay pathways that can occur. A model of the possible pathways for energy and electron transfer is shown in Figure S6. Identifying bottlenecks in these pathways could be an important next step in the development of biophotovoltaics. Overall, we can surmise that (i) the TR chromophore is effective as an excitation energy donor to LHCII and this enhances the photocurrent generated, (ii) this TR enhancement effect is limited by some combination of LHCII/TR self-quenching, surface-related quenching and/or an inherent limitation in the rate of electron transfer to/from LHCII. One recent study^47^ attempted to quantify the energy loss pathways in a biohybrid photovoltaic system involving RC and LH complexes by using a combined instrument for spectroscopy and electrochemistry measurements in situ (on electrodes) and this technique could be useful for further optimizing the performance of future devices.

### 3.5 Future Outlook for the Utility of LH Protein Complexes to Generate Photocurrent

Our previous proof-of-concept research highlighted the effectiveness of chromophores localized in lipid bilayer for broadening the absorption range of LH proteins.^26−29^ The findings presented in the current study build upon the idea, by demonstrating the application of this concept to photovoltaic devices. While the photocurrent production achieved by this LH antenna complex-based system is lower than what can be achieved with naturally electron-transferring RC proteins alone,^23,48^ LH proteins have the potential to enhance the performance of RC proteins too, so it may be possible to use combinations of RCs, LHCs and synthetic chromophore molecules.

The present architecture using the self-assembling lipid bilayer system exhibited an efficient flow of excitation energy from TR to LHCII Chls, which leads to photocurrent generation reaction in LHCII. We speculate that the terminal Chl *a* 610–611, close to the stromal side of LHCII, act as photocatalytic pigments.^13,14^ After LHCII is excited, a molecule of the mediator MV would bind to some site on LHCII and accept an electron from the excited Chls, generating the oxidized LHCII (LHCII^+^), which would then accept an electron from the electrode surface (Figure S6). The direct electron transfer (DET) from the electrode to the active Chl is thought to be a rate-limiting step, for which an LHCII protein orientation where the stromal side faces the electrode would be preferable for the DET. However, the hydrophilic MV may have less access to the active Chl for the LHCII with the orientation of stromal side facing the electrode, if the MV binding site is also located close to the stromal side. For LHCII with the opposite direction, the accessibility of MV to the active Chl would be higher but direct electron transfer would be less favorable. In this way, either orientation of LHCII within the lipid bilayer could generate photocurrent but their activity may vary. Future work could characterize which orientation of LHCII is preferable and quantify the fraction of “active LHCII”^21−24^ (methods to control the orientation of LHCII could then be developed^49^). In addition, the amount of the active LHCII on the electrode will directly influence the photocurrent value. In future work, assembling LHCII (and TR) onto an electrode with a larger surface area, such as inverse opal ITO, could provide larger photocurrents.^50^ The simplest conceivable interaction between the membranes and the electrode would be a single lipid bilayer (containing LHCII and TR-DHPE) on the ITO surface, but multilayers of lipid bilayers would be possible due to the electrostatic interactions that can occur between LHCII.^51^ There can only be direct electron transfer from the ITO substrate to LHCII within the first lipid bilayer, but excitation energy transfer (FRET) can proceed over large distances between neighboring LHCII.^52^ Furthermore, the electron mediator methyl viologen is small enough to diffuse between layers because there will be space for lateral diffusion between the first and any subsequent layers and large interlayer cavities. In this way, multiple layers can support the generation of photocurrent.

A number of natural or seminatural photosynthetic systems have previously been demonstrated to (or have the potential to) convert solar energy into electrical current when interfaced with an electrode. We suggest that complementary chromophores could be incorporated to enhance the effectiveness of such biohybrid solar cells, by following our approach. Popular biohybrid systems include RC proteins in mono- or multilayer films deposited onto electrodes.^2,50,53,54^ These can be combined with other natural photosynthetic components, such as LH proteins to further enhance energy capture,^15,21^ or cytochrome complexes to act as natural electron mediators.^55−57^ Instead of the minimalist approach of using isolated photosynthetic proteins, as used in the current study, it is also possible to generate functional devices using intact natural plant membranes, such as extracted thylakoid membranes that are still capable of oxygen evolution in addition to electron transfer from both photosystems^58^ or even to extract electrons from living cells, such as cyanobacteria.^59,60^ A prerequisite for combining complementary chromophores with more complex natural systems would be selecting appropriate chromophores that naturally localize with the biological components through self-assembly. It has previously been shown that the chemistry of the chromophore (lipid-linked, lipophilic, etc.) can have a significant effect on the orientation that it incorporates into the lipid-bilayer and where the photoactive group is located.^26,29,61^ The orientation of the chromophore, and other factors such as chromophore–protein attraction, will have a significant influence on the effectiveness of the chromophore in addition to the spectral properties.^62,63^ A potential future extension of this concept would be the spectral enhancement of whole cells deposited onto electrodes. It has previously been demonstrated that cyanobacteria cells, either as a single layer or as a multilayer biofilm, are capable of producing electrical current under photo illumination.^59,64^ While the level of photocurrent generation reported is lower for cyanobacteria compared to using highly concentrated extracts of thylakoid membranes, intact biological cells have the aforementioned advantage of significantly higher long-term stability. Incorporating complementary chromophores into cells could potentially be achieved by electroporation where the permeability of cells is temporarily increased allowing the uptake of non-native material.^65^

## 4 Conclusions

In this study, we successfully demonstrated the application of lipid-linked TR chromophores in conjunction with LH antenna complexes from plants for enhancing the amount of photocurrent generated in photovoltaic devices. Instead of using RC-type complexes, which are commonly employed in constructing biohybrid photovoltaic devices with natural photosynthetic material, LH complexes were found to act as a viable alternative and organic chromophores improved their effectiveness. This was possible by the somewhat surprising ability of the plant LHCII to donate electrons when incorporated into a photovoltaic device, as discovered in a previous study.^13^ The LH complexes were held in place by a lipid bilayer, providing a more stable orientation compared to LH complexes stabilized by detergents, as seen in previous research. The TR chromophore was integrated into the lipid bilayer at a range of concentrations and was shown to effectively donate excitation energy to the LHCII, thereby expanding the spectral absorption range of the LH complexes and facilitating greater photocurrent production. However, there was a limit to the enhancement effect of the additional energy transfer pathway, as demonstrated by a saturation of TR-related photocurrent production even when increasing TR concentration relative to LHCII on electrodes. Quenching effects related to either the protein, lipid, or the substrate itself appear to be involved. This research further expands on the concept of enhancing photosynthetic proteins with synthetic chromophores and demonstrates its potential advantages when applied to biohybrid photovoltaic devices. The extension of this concept to more complex photosynthetic systems or even whole cells deposited onto electrodes offers a promising avenue for future research.

## Data Availability

All relevant raw and analyzed data associated with this paper are openly available under a CC-BY license in the Research Data Leeds repository^66^ and can be found at doi: 10.5518/1621

## Supporting Information Available

Comparison of the spectra of LHCII within detergent micelles versus LHCII within liposomes by absorption and fluorescence spectroscopy (Figure S1); analysis of fluorescence excitation spectra of LHCII-TR proteoliposomes to assess the FRET efficiency (Figure S2); further analysis of action spectra comparing raw versus normalized data (Figure S3); analysis of the quenching of TR by fluorescence spectroscopy (Figure S4); analysis of the photocurrent generated in the absence of the LHCII protein complex (Figure S5); schematic showing the network of transitions that could occur between TR, LHCII, MV, and the electrodes (Figure S6); calculation of the concentration of TR and LHCII found in the liposomes as prepared (Table S1); quantification of the amount of material deposited on ITO electrodes coated with the LHCII/DOPG/TR-DHPE membranes (Table S2); calculation of the efficiency of TR-to-LHCII excitation energy transfer at various TR-to-LHCII ratios (Table S3); observed photocurrents from the LHCII/DOPG(/TR-DHPE) membranes on ITO electrodes (Table S4); calculation of the fluorescence lifetimes from the fitting of streak camera data of TR/DOPG liposomes in solution (Table S5); calculation of the fluorescence lifetimes from the fitting of streak camera data of TR/DOPG SLBs on ITO electrode surfaces (Table S6) (PDF)

## Abbreviations

β-DDM: *n***-**dodecyl-β-d-maltoside
β-OG: *n*-octyl-β-d-glucopyranoside
Car: carotenoid
Chl: chlorophyll
DOPG: 1,2-dioleoyl-*sn*-glycero-3-phospho-(1′-*rac*-glycerol)
FRET: Förster resonance energy transfer
ITO: indium tin oxide
LH: light-harvesting
LHCII: light-harvesting pigment–protein complex II
MV: methyl viologen
MLV: multilamellar vesicle
PSI/PSII: photosystem I/II
RC: reaction centre
SAM: self-assembled monolayer
SLB: supported lipid bilayer
TR: Texas Red
TR-DHPE: Texas Red dihexadecanoyl-*sn*-glycero-3-phosphoethanolamine
Tris: tris(hydroxymethyl)aminomethane
