# Supplementary material for: Photocurrent Generation by Plant Light-Harvesting Complexes is Enhanced by Lipid-Linked Chromophores in a Self-Assembled Lipid Membrane
Source: J Phys Chem B. 2025 Jan 9;129(3):900–10. doi: 10.1021/acs.jpcb.4c07402 (PMC11770764; doi:10.1021/acs.jpcb.4c07402)
Supplement: Supplementary file 1 — jp4c07402_si_001.pdf [file jp4c07402_si_001.pdf]

Supporting Information:

Photocurrent Generation by Plant Light-Harvesting Complexes is Enhanced by Lipid-Linked Chromophores in a Self-Assembled Lipid Membrane

Masaharu Kondo,[1,*] Ashley M. Hancock,[2,3] Hayato Kuwabara,[1]
Peter G. Adams,[2,3,*] and Takehisa Dewa[1,4,*]

1: Department of Life Science and Applied Chemistry, Graduate School of Engineering, Nagoya Institute of Technology, Gokiso-cho, Showa-ku, Nagoya 466-8555, Japan

2: School of Physics and Astronomy, University of Leeds, Leeds LS2 9JT, U. K.

3: Astbury Centre for Structural Molecular Biology, University of Leeds, Leeds LS2 9JT, UK

4: Department of Nanopharmaceutical Sciences, Nagoya Institute of Technology, Gokiso-cho, Showa-ku, Nagoya 4668-8555, Japan

*for correspondence:
kondo.masaharu@nitech.ac.jp
p.g.adams@leeds.ac.uk
takedewa@nitech.ac.jp

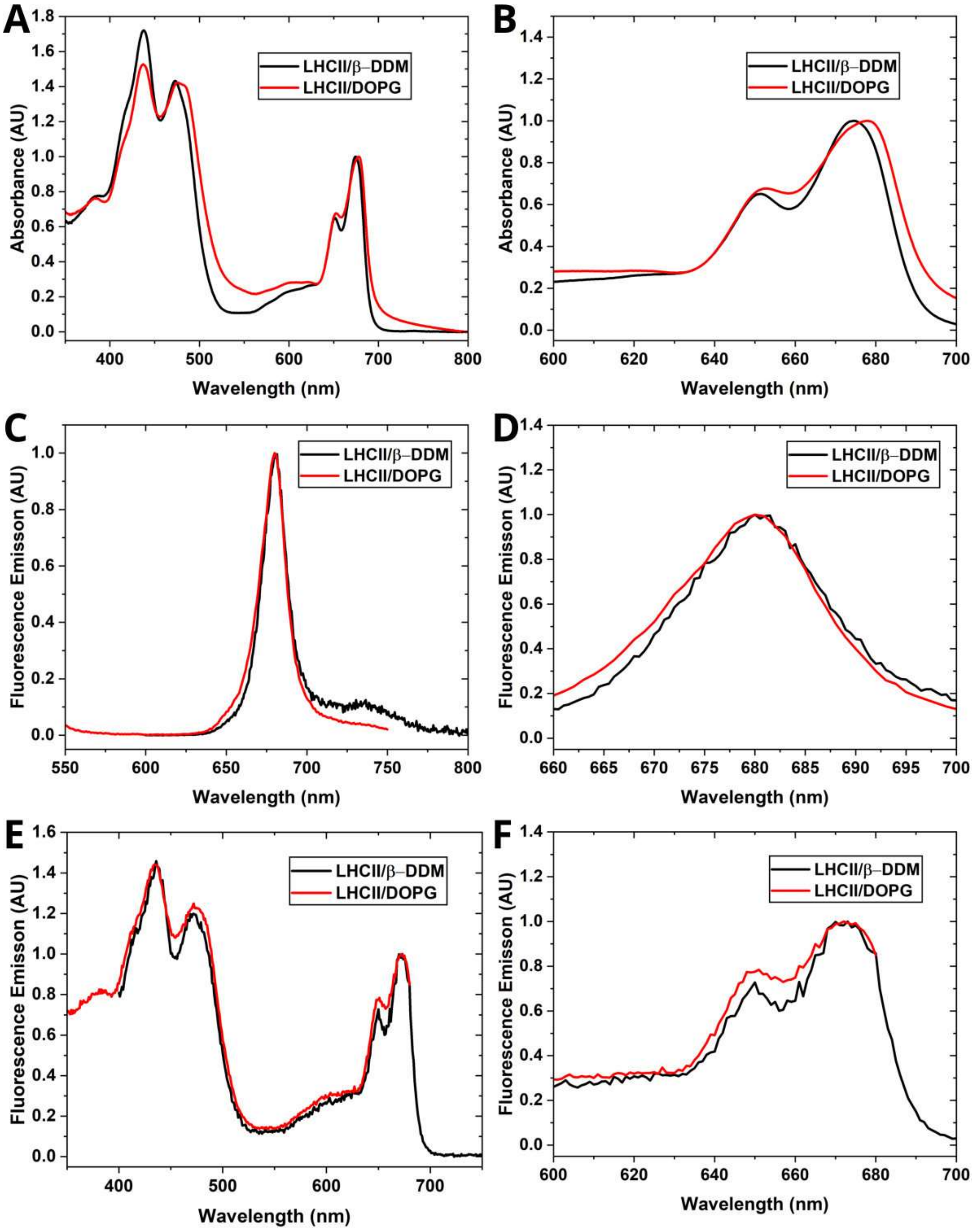

Figure S1. Comparison of the spectra of LHCII within detergent micelles versus LHCII within liposomes. **(A)**, **(B)** Absorption spectra. There is a subtle red-shift in the chlorophyll Q_y peak of ~5 nm which could be due to the influence of light scattering. The ratio of the peaks appears to be similar. **(C)**, **(D)** Fluorescence emission spectra upon excitation at 438 nm. There is no observable shift in the peak maxima. **(E)**, **(F)** Fluorescence excitation spectra monitored at 685 nm. There are no significant spectral shifts in the excitation spectrum which reduced any concerns about the absorption spectra (suggesting the shift in panels A-B was indeed due to light scattering). The LHCII was either stabilized by the detergent β-DDM (black plots) or reconstituted into proteoliposomes (red plots).

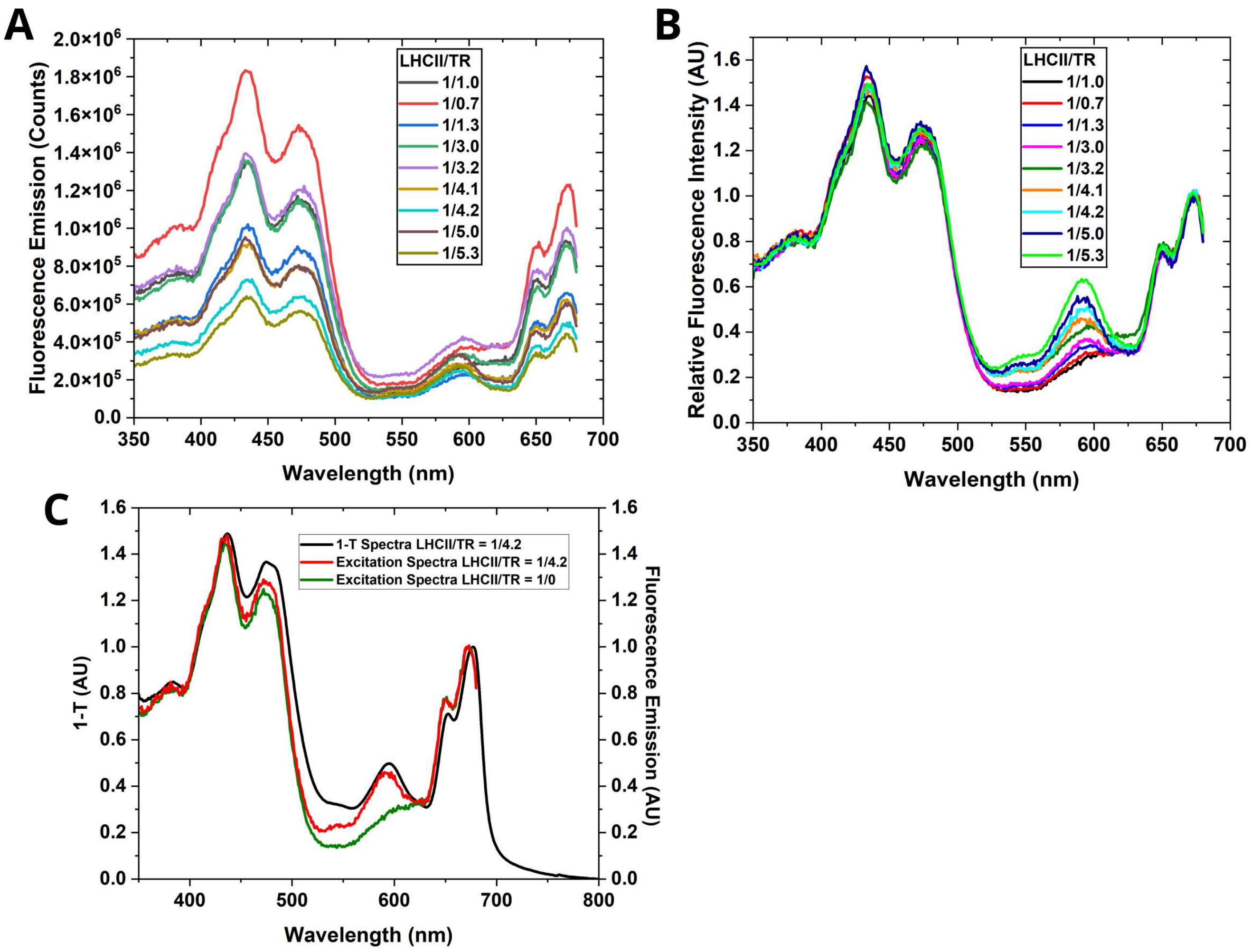

Figure S2. Analysis of fluorescence excitation spectra of LHCII-TR proteoliposomes in solution to assess the FRET efficiency. Raw **(A)** and normalized data **(B)** of the fluorescence excitation spectra of proteoliposomes in solution, normalized to a height of 1.0 at 675 nm (the chlorophyll *a* Q_y band). **(C)** Example of how these excitation spectra were compared with absorptance (1 – T) spectra to calculate FRET efficiency. FRET efficiency is shown in **Table S3**.

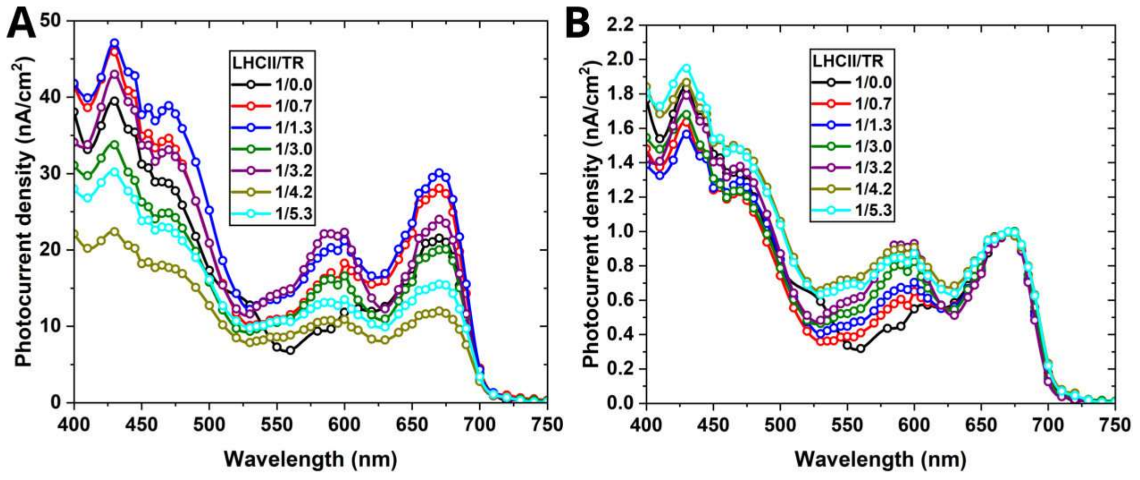

Figure S3. Further analysis of action spectra comparing raw versus normalized data. Raw data **(A)** and normalized data **(B)** of the action spectra of selected devices containing LHCII-TR membranes on ITO electrodes, normalized to a height of 1.0 at 675 nm.

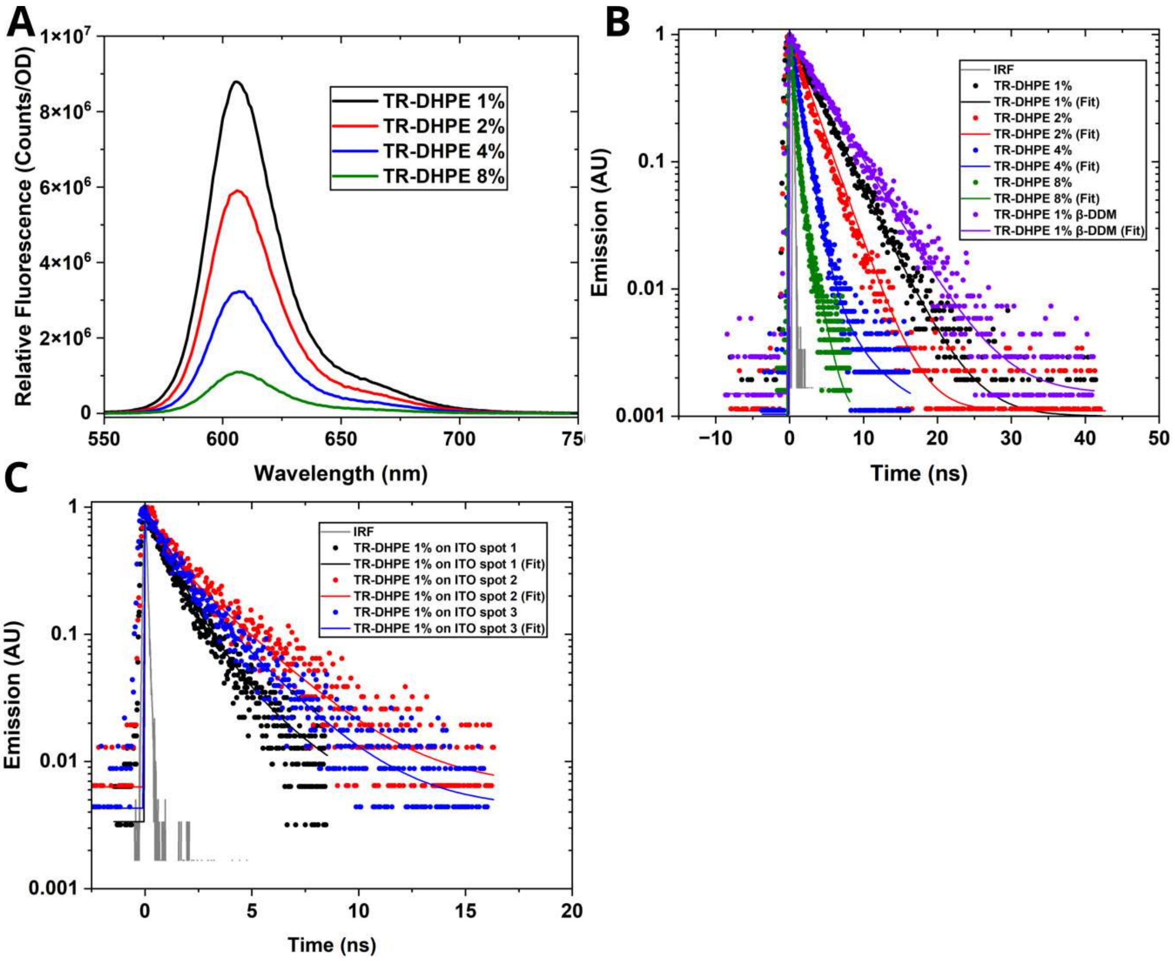

Figure S4. Analysis of the quenching of TR by fluorescence spectroscopy. **(A)** Fluorescence emission spectra of TR-DHPE/DOPG liposomes in solution. Sample concentration was adjusted an absorbance of 0.1 at 591 nm in a quartz cell with 10 mm of path length and the raw data is shown, revealing the relative decrease in fluorescence intensity for the same TR concentration. **(B)** Time-resolved fluorescence data of the same samples from (A). The finding that the exponential decay of fluorescence becomes increasingly steep is consistent with TR quenching. The fluorescence lifetime values calculating after exponential fitting are shown in **Table S5**. **(C)** Time-resolved fluorescence data of TR-DHPE/DOPG SLBs on ITO surfaces. The fluorescence lifetime values calculating after exponential fitting are shown in **Table S6**. Time resolved measurements were acquired with a streak camera monitoring the fluorescence emission at 610 nm, which represents the fluorescence decay of TR. All samples were measured in 50 mM Tris-HCl buffer (pH 7.4).

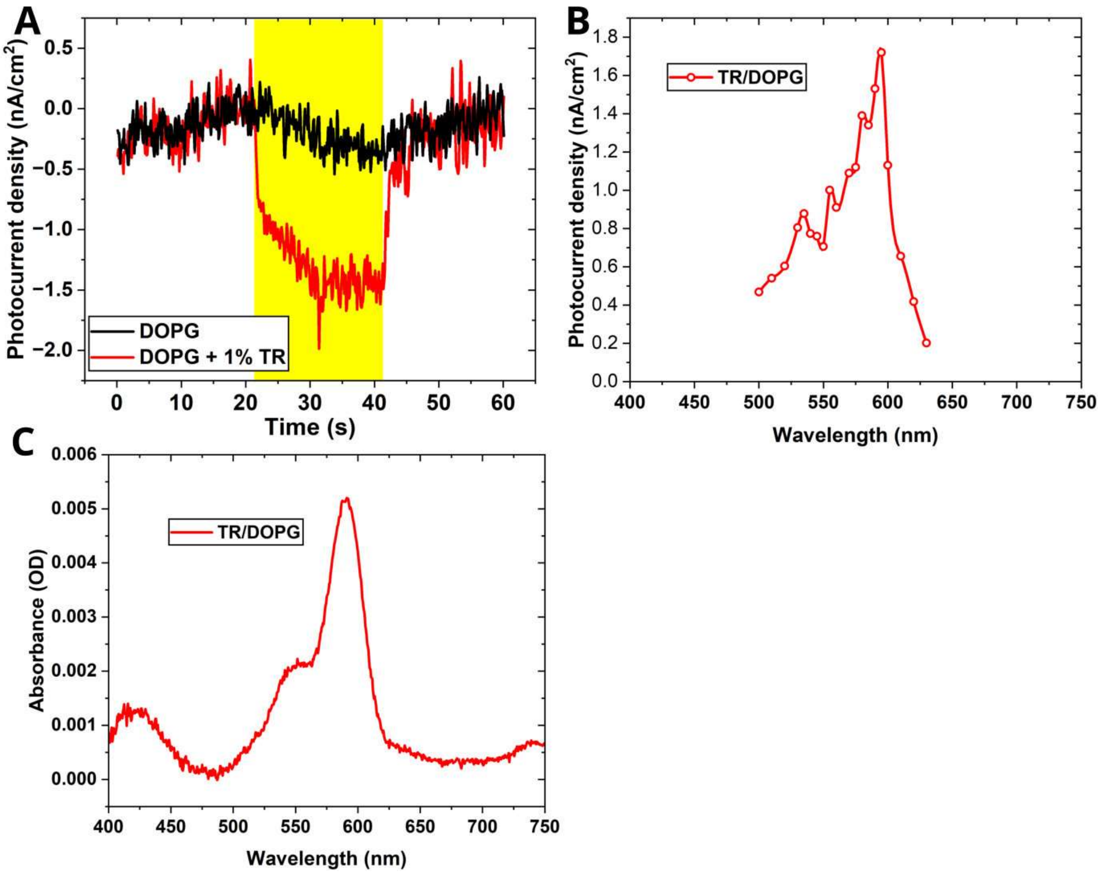

Figure S5. Analysis of the photocurrent generated in the absence of the LHCII protein complex. **(A)** Photocurrent response of membranes generated on 6-amino-hexanethiol modified ITO electrodes, using the liposomes comprised of 1/100 TR-DHPE/DOPG (red) or 100% DOPG (black). The photocurrent was monitored at 590 nm. **(B)** Photocurrent action spectrum and **(C)** in situ absorption spectrum of the TR-DHPE/DOPG membranes on ITO sample. The electrolyte solution was comprised of 0.1 M phosphate buffer (pH 7.5), 0.1 M $NaClO_4$ and 10 mM methyl viologen. From a peak absorbance of 0.0052 at 590 nm the amount of TR deposited onto the electrode was calculated as 44.8 pmol/cm^2 (using a molar absorption coefficient of 116,000 M^{-1} cm^{-1}).

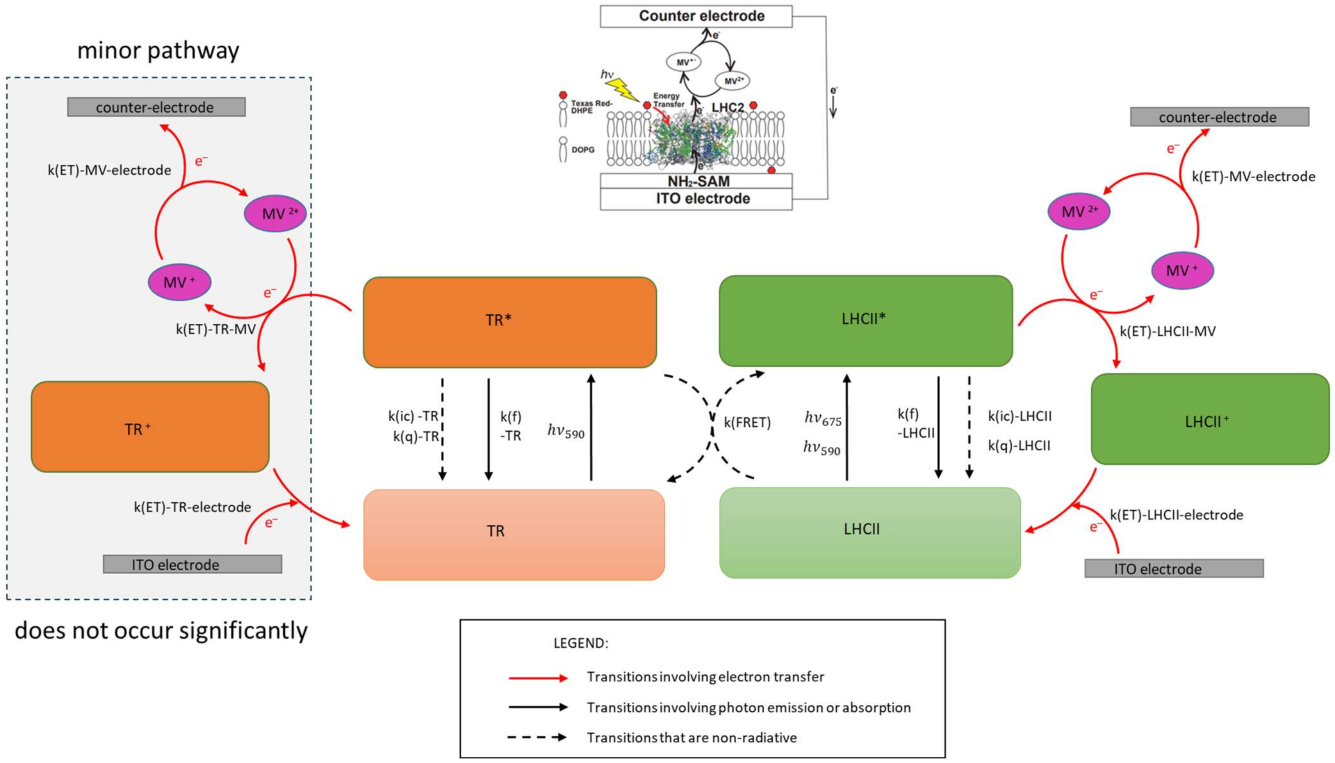

Figure S6. Schematic showing the network of transitions that could occur between TR, LHCII, MV and the electrodes. This schematic represents a possible kinetic model (not a Jablonski diagram or a structural model). TR/TR*/TR^+ and LHCII/LHCII*/$LHCII^+$ represent the ground state/ excited state/ cationic state. hν = photon absorption (considered instantaneous, sub-picosecond); k(f) = rate of fluorescence; k(ic) = rate of internal conversion; k(q) = rate of decay due to quenching pathways, (e.g., self-quenching); k(ET) = rate of electron transfer; k(FRET) = rate of excitation energy transfer (from TR to LHCII). We could treat all **k** as inherent rates (i.e., constant). In that case, we would need to consider that the overall number of transfers depend on the absolute concentrations, i.e., we could introduce terms for C(TR) and C(LHCII). Or we could treat all **k** as the overall rates that include concentration and therefore the rates would change. This kinetic scheme illustrates the photophysical complexity of the system and cannot be solved in the current work.

Table S1. Calculation of the concentration of TR and LHCII found in the liposomes as prepared.

TR abs (590nm)	TR abs* (590nm)	TR conc (µM)	LHCII abs (Chl *a* peak, 675 nm)	LHCII abs (Chl *a* peak, 675 nm)*	LHCII conc (µM)	TR:LHCII ratio# (mol/mol)
0.000	0.00	0.00	0.087	4.44	2.61	0.0
0.006	0.33	2.84	0.069	3.54	2.08	1.4
0.014	0.71	6.14	0.099	5.04	2.97	2.1
0.019	0.97	8.37	0.098	5.02	2.95	2.8
0.025	1.25	10.81	0.105	5.37	3.16	3.4
0.024	1.24	10.69	0.102	5.22	3.07	3.5
0.031	1.59	13.74	0.092	4.67	2.75	5.0
0.032	1.65	14.20	0.084	4.30	2.53	5.6
0.039	1.98	17.05	0.101	5.13	3.02	5.6

Abs: measured absorbance; conc: concentration;
*: value for absorbance after correction for 1/51 dilution and deconvolution;
For LHCII, the peak height related to chlorophyll *a* at 675 nm was measured.
Molar absorption coefficients used for concentration calculation were: 1,700,000 M^{-1} cm^{-1} (at 675 nm) for LHCII trimers and 116,000 M^{-1} cm^{-1} (at 590 nm) for TR.
#: ratio calculated from the concentration values calculated for LHCII and TR shown in this table.

Table S2. Quantification of the amount of material deposited on ITO electrodes coated with the LHCII/DOPG/TR-DHPE membranes.

TR abs (590nm)	TR conc (pmol/cm^2)	LHCII abs (Chl *a* peak, 675 nm)	LHCII conc (pmol/cm^2)	TR:LHCII on ITO* (mol/mol)	TR:LHCII in liposomes$^\#$ (mol/mol)
0.000	0.00	0.064	37.53	0.0	0.0
0.002	14.26	0.036	21.00	0.7	1.4
0.005	46.60	0.062	36.75	1.3	2.1
0.004	31.50	0.018	10.57	3.0	2.8
0.006	48.72	0.026	15.45	3.2	3.4
0.008	69.76	0.029	16.88	4.1	3.5
0.011	90.91	0.037	21.85	4.2	5.0
0.004	35.44	0.012	7.03	5.0	5.6
0.017	145.43	0.047	27.48	5.3	5.6

Abs: measured absorbance; conc, concentration;
For LHCII, the peak height related to chlorophyll *a* at 675 nm was measured.
Molar absorption coefficients used for concentration calculation were: 1.7×10^{-3} pmol^{-1} cm^{-2} for LHCII trimers and 1.16×10^{-4} pmol^{-1} cm^{-2} for TR.
*: ratio calculated for membranes on ITO using the concentrations for LHCII and TR shown in this table.
$^\#$: ratio for the corresponding proteoliposome samples, shown for comparison (repeated from **Table S1**).

Table S3. Calculation of the efficiency of TR-to-LHCII excitation energy transfer at various TR-to-LHCII ratios.

Ratio of TR/ LHCII	Energy Transfer Efficiency
0.0	N/A
0.7	0.86
1.3	0.88
3.0	0.93
3.2	0.92
4.1	0.88
4.2	0.87
5.3	0.84

The energy transfer efficiency was calculated by comparing the absorptance spectra (1 – T) to the fluorescence excitation spectra, as shown in **Figure S2C**. This data shows that the FRET efficiency is similar as the amount of TR varies (this is concurrent with previous work that as showed the efficiency varies with the FRET acceptor concentration and is not affected by the FRET donor concentration. Therefore, when the amount of TR increases whilst the FRET efficiency is relatively consistent this will lead to an increase in the overall magnitude of energy transferred to the LHCII.

Table S4. Observed photocurrents from the LHCII/DOPG(/TR-DHPE) membranes on ITO electrodes. This data was used to plot the graph in the main text **Figure 4D**.

[a] Ratio of TR/ LHCII	[b] Photocurrent at 590 nm [nA/cm^2]	[b] Photocurrent at 675 nm [nA/cm^2]	[c] Ratio of photocurrents (590/675nm)
0.0	9.7	21.0	0.46
0.7	17.0	27.4	0.62
1.3	20.3	29.5	0.69
3.0	22.1	23.5	0.94
3.2	10.8	11.7	0.92
4.1	3.2	3.6	0.89
5.3	13.1	15.4	0.85

[a] Calculated from absorption spectra, as shown in **Table S2**.
[b] Measured from photocurrent action spectra in **Figure 4A/ Figure S3**.
[c] Calculated from the two preceding columns.

Table S5. Calculation of the fluorescence lifetimes from the fitting of streak camera data of TR/DOPG liposomes in solution, from Figure S4B.

Sample	τ1 [ns]	τ2 [ns]	Amp1	Amp2	τ av. [ns]
1% 0.03 wt% β-DDM	4.40	-	100	-	4.40
1%	3.60	-	100	-	3.60
2%	2.65	-	100	-	2.65
4%	4.20	1.16	2.3	97.7	1.23
8%	1.31	0.390	11.5	88.5	0.497

Table S6. Calculation of the fluorescence lifetimes from the fitting of streak camera data of TR/DOPG SLBs on ITO electrode surfaces, from Figure S4C.

1% ITO Sample	τ1 [ns]	τ2 [ns]	Amp1	Amp2	τ av. [ns]
Spot1	2.83	1.12	15.0	85.0	1.37
Spot2	2.76	0.664	54.5	45.5	1.81
Spot3	2.52	0.950	46.9	53.1	1.68
